# Physicochemical Characterization and *In Vitro* Anti-Inflammatory Assessment of Novel Sodium Alginate Sponges Loading *Andiroba* Oil (*Carapa guianensis* Aubl.) for Skin Dressings

**DOI:** 10.1021/acsomega.5c10665

**Published:** 2026-02-04

**Authors:** Marinaldo V. de Souza Junior, Jad Lorena F Simplicio, Fernanda F. Costa, Aramys S. Reis, Eliana B. Souto, Adenilson O dos Santos, Francisco F. de Sousa

**Affiliations:** † Center for Sciences of Imperatriz, Federal University of Maranhão—UFMA, 65900-410 Imperatriz, MA, Brazil; ‡ UCD School of Chemical and Bioprocess Engineering, 8797 University College Dublin, Belfield, Dublin 4 D04 V1W8, Ireland; § Laboratory of Pathophysiology and Therapeutic Research, Center for Social Sciences, Health and Technology, Federal University of Maranhão, Imperatriz, MA 65900-410, Brazil; ∥ Institute of Exact and Natural Sciences, Federal University of Pará—UFPA, 66075-110 Belém, PA, Brazil

## Abstract

This study describes the structural characterization of a novel formulation based on sodium alginate sponges loading the Amazonian *andiroba* oil (*Carapa guianensis* Aubl.) as a strategy for developing topical anti-inflammatory dressings. The characterization by X-ray diffraction revealed the amorphous profile of unloaded and *andiroba* oil-loaded sponges, indicating structural flexibility suitable for modifying the release profile of bioactives. Scanning electron microscopy revealed a porous, interconnected structure of the unloaded alginate sponges, whereas oil-loaded sponges exhibited smoother, thicker pore walls and localized densification, indicating the oil’s influence on the polymeric matrix architecture. Fourier transform infrared spectra identified the ester, hydroxyl, and carboxylate groups, confirming the chemical signature of the *andiroba* oil and its interactions with the alginate matrix. Hirshfeld surface analysis of oleic acid, one of the main bioactive components of *andiroba* oil, revealed a predominance of hydrophobic contacts and hydrogen-bonding interactions, supporting its affinity for biological targets. Surface analysis also indicated high porosity (∼70% void volume), suggesting a potential application for topical delivery. Docking simulations showed favorable binding affinity of oleic acid to the active site of NF-kB (−5.75 kcal/mol) and iNOS (−5.15 kcal/mol), corroborating its anti-inflammatory potential. *In silico* pharmacokinetic profiling exhibited low skin permeation (Log *K*_p_ = −2.6 cm/s) of oleic acid, no blood–brain barrier penetration, and no interaction with P-glycoprotein. *Andiroba* oil-loaded sponges significantly reduced nitric oxide production in lipopolysaccharide-activated RAW 264.7 macrophages without affecting cell viability. The unloaded alginate sponges also showed mild nitric oxide inhibition at high concentration, confirming their inherent biocompatibility. Altogether, these findings support the use of the developed sponges as a promising bioactive dressing for the healing of inflamed wounds and other chronic skin conditions, combining Amazonian phytotherapy with innovative polymeric delivery systems.

## Graphical abstract

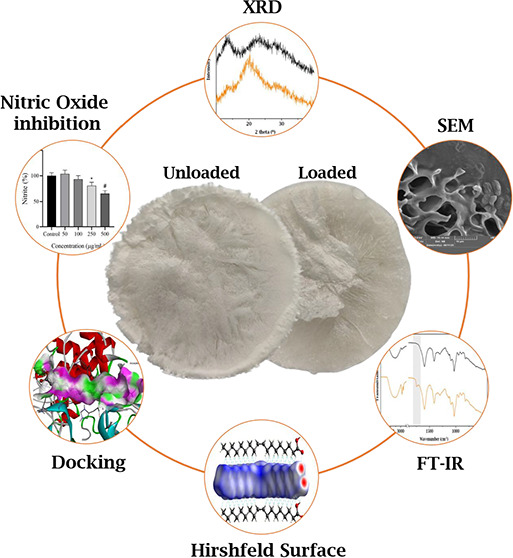

## 1 Introduction

Inflammation is an essential physiological response to tissue injury, mediated by a cascade of molecular signals involving the production of reactive species, such as nitric oxide, as well as pro-inflammatory mediators, including prostaglandins and cytokines.^1–3^ When dysregulated or chronic, this process can compromise healing and promote drug resistance, leading to persistent wounds and the development of complex inflammatory diseases.^4–6^ In this context, there is growing interest in therapeutic alternatives with local anti-inflammatory action, especially those of natural origin, which can modulate the inflammatory response efficiently and with less toxicological risk.

Among the phytotherapeutic resources of Amazonian biodiversity, *andiroba* oil (*Carapa guianensis* Aubl.), a member of the Meliaceae family, stands out for its traditional use in treating inflammation, joint pain, insect bites, and other skin conditions.^7,8^ This oil is rich in a variety of bioactive compounds, such as limonoids, triterpenes, and unsaturated fatty acids, mostly in oleic acid.^8–10^ Despite its therapeutic potential, the pharmaceutical application of *andiroba* oil still faces challenges, mainly related to its low solubility in aqueous media, oxidative instability, and difficulty in delivery by conventional topical dosage forms.^11,12^ Alternatively, the use of natural biomaterials as modified-release vehicles has proven to be an effective strategy for overcoming these limitations, while simultaneously enhancing the biological activity of *andiroba* oil.^13,14^

In the group of natural polymers, sodium alginate, a polysaccharide extracted from brown seaweed, exhibits broad biocompatibility, biodegradability, and excellent moisture retention capacity.^15–17^ In the form of sponges or hydrogels, alginate has been widely used in wound dressings due to its ability to absorb exudate, promote adequate moisture in the wound bed, and serve as a matrix for modified drug release.^13,18–20^ The incorporation of lipophilic compounds, such as *andiroba* oil, into the alginate matrix requires a detailed investigation of their influence on the structural organization, physicochemical stability, and biological effects of the resulting biomaterial.

Previous studies have demonstrated that the introduction of hydrophobic substances into hydrophilic matrices can directly affect crystallinity, molecular interactions, and the distribution of active ingredients within the polymeric biomaterial.^13,21,22^ Therefore, investigations using X-ray diffraction (XRD) and Fourier transform infrared (FT-IR) spectroscopy become essential for assessing the presence of specific interactions, possible conformational changes, and the degree of structural organization of the biomaterial. XRD analysis was used by Simplício et al.^13^ to describe the formation of amorphous structures after incorporating *andiroba* oil into sodium carboxymethyl cellulose sponges. The obtained biomaterial was characterized by the absence of well-defined crystalline peaks, indicating a beneficial disorganization of the crystalline structure that facilitates the formation of multiple micropores irregularly distributed in the polymeric matrix. Furthermore, FT-IR spectra confirmed not only the presence of the functional groups characteristic of *andiroba* oil but also vibrational bands typical of fatty acids, namely, palmitic and linoleic acids.

Given the above, this study proposes an integrated approach to investigate the impact of incorporating *andiroba* oil in sodium alginate sponges, with an emphasis on the correlation among physicochemical characteristics, structural properties, and anti-inflammatory activity. Complementary analyses were performed, *i.e*., FT-IR to identify the vibration bands of main functional groups, XRD to assess crystallinity, and scanning electron microscopy (SEM) to record the matrix’s morphology. Advanced computational methods, such as Hirshfeld surface analysis and molecular docking modeling, using oleic acid as a representative marker of the lipophilic fraction of *andiroba* oil, corroborated the results of the *in vitro* assays. A significant reduction in nitric oxide release by macrophages stimulated with lipopolysaccharide (LPS) was seen when treated with *andiroba* oil-loaded sponges without compromising cell viability, as shown by cytotoxicity tests.

This integration of experimental and *in silico* methods allows for a more comprehensive understanding of how the molecular structure of bioactives influences their pharmacological performance in biomaterials. The proposed incorporation of *andiroba* oil into sodium alginate sponges not only expands its therapeutic applications but also represents a breakthrough in the valorization of resources from Amazonian biodiversity, combining technological innovation with sustainability. The results presented here will significantly contribute to the development of dressings based on natural active ingredients with potential applications in the treatment of topical inflamed lesions and other chronic skin diseases.

Compared to the work of Simplício et al.,^13^ which primarily focused on the physicochemical characterization of Cu­(II)-complex sponges, the present study introduces a biologically driven perspective by employing a biocompatible alginate matrix and integrating *in vitro* anti-inflammatory assays with advanced molecular modeling. This combination provides mechanistic and functional insights that have not been previously investigated.

## 2 Materials and Methods

### 2.1 Materials

*Andiroba* oil was sourced in its natural state from a local market (registered in SisGen under the number A05C495). Unless otherwise specified, all other materials and reagents were purchased from Sigma-Aldrich (St. Louis, MO, USA). Deionized water was obtained from a home-supplied system using a Permution deionizer (Permution, Curitiba, PR, Brazil).

### 2.2 Formulation of Sponges

Two solutions containing 1 g of sodium alginate (MW = 198.00 g/mol) dissolved in 50 mL of deionized water were prepared under stirring until complete solubilization of the polymer. One of these solutions was used as a control, while the other was added with 1.96% *andiroba* oil (∼0.02 g). This solution was selected based on optimization studies^13,14,23^ reported for similar oil-loaded polymeric systems, where lipid fractions within 1–3% interval ensured structural homogeneity and mechanical integrity of the sponge. Both were kept under continuous stirring at 300 rpm for 24 h (VELP Scientifica, Usmate, Italy), and then transferred to Petri dishes, to be cooled down to −4 °C for 24 h. They were then lyophilized in a TERRONI LS3000 lyophilizer (Lyotech, São Carlos, SP, Brazil) at −40 °C for another 24 h, resulting in unloaded and *andiroba* oil-loaded sponges for later analysis.

### 2.3 Experimental Characterization Techniques

To investigate the structure of the obtained polymeric sponges, X-ray diffraction (XRD) analysis was performed using a PANalytical Empyrean diffractometer (Malvern PANalytical, Malvern, United Kingdom). The equipment operated with Cu–K_α_ radiation (λ = 1.5418 Å) under 40 kV and 40 mA conditions. Measurements were performed at room temperature, covering the angular range from 5 to 50° (2θ), with 0.02° steps every 2 s.

The morphology of the sponges was analyzed by scanning electron microscopy (SEM) using a Tescan Vega3 SB instrument (Kohutovice, Czech Republic). The samples were fixed to carbon tape and coated with a thin layer of gold/palladium (20 nm) to improve the electrical conductivity. Images were obtained at low accelerating voltages (between 6 and 10 kV) to preserve the sample integrity. Micrographs were recorded at a magnification scale of 10 μm to detail the surface morphology. Elemental composition was evaluated by energy-dispersive X-ray spectroscopy (EDS) using a detector coupled to an SEM instrument. Spectra were acquired from representative regions of the sponge surface under the same operating conditions used for the imaging.

The percentage of porosity and mean pore diameter were determined from SEM micrographs using ImageJ software (NIH, Bethesda, USA).^24,25^ Images were calibrated with the 10 μm scale bar, converted to grayscale, and binarized to quantify the pore area. Porosity was calculated as the ratio between the total pore area and the analyzed image area following standard image analysis procedures.

Fourier transform infrared (FT-IR) spectroscopy was used to identify the functional groups present in the sponges. Measurements were performed in transmission mode using a Bruker Vertex 70 V spectrometer (Bruker, Billerica, Massachusetts, USA), covering the spectral range of 400 to 4000 cm^–1^. For this purpose, the ATR A225/Q Platinum Total Reflectance Attenuated module was used, together with a wide-aperture (6 mm) RT-DLA TGS detector, allowing measurements from 400 cm^–1^ with a spectral resolution of 4 cm^–1^ in 100 scans.

### 2.4 Computational Methodology

Hirshfeld surfaces of the oleic acid, the selected *andiroba* oil biomarker, were obtained using Crystal Explorer 17 software,^26^ based on the crystallographic information file (.CIF) deposited under number 1226004 at the Cambridge Crystallographic Data Centre (CCDC). This approach allowed us to investigate the intermolecular contacts of the biomarker. The three-dimensional (3D) Hirshfeld surfaces of the oleic acid were mapped based on three structural properties: normalized distance (*d*_norm_), shape index, and curvedness.^27^ Furthermore, voids were identified using electron density isosurfaces with a value of 0.002 au, as proposed by Bader et al.,^28^ allowing visualization of the free spaces in the unit cell and a better understanding of the structural organization of the fatty acid.

Molecular docking of oleic acid was performed with different proteins belonging to the immune system. The following crystal structures of the protein models used as receptors were downloaded from the Protein Data Bank:^29^ iNOS (PDB code 6DN6) and IkBα/NF-kB transition factor (PDB code 1IKN). The files autodock4.exe, autogrId4.exe, and AD4.1_bound.exe were separated into a folder along with the .PDB files of the proteins and oleic acid. Then, proteins were prepared by removing the cocrystallized ligand, free water molecules, and cofactors, leaving only the residues associated with the proteins. Grid was defined to surround the region of interest in the macromolecules. Molecular docking calculations were performed using the computational packages AutoDock Vina and AutoDockTools (version 1.5.7)^30^ and the Discovery Studio viewer. The results were classified according to the values of binding free energy, ligand efficiency, and inhibition constant (*K*_i_) obtained in docking calculations.

Docking reliability was checked through a redocking validation step using the cocrystallized ligands of NF-kB and iNOS as reference compounds. The RMSD values obtained for the best-ranked poses were below 2.0 Å, confirming the accuracy of the docking protocol. The grid box was centered on the active-site residues of each target and adjusted according to the molecular dimensions of the binding pockets. For docking simulations with the NF-kB p65 subunit (PDB ID: 1IKN), the grid box was defined with dimensions of 126 × 120 × 126 Å, whereas for iNOS (PDB ID: 6DN6), it was set to 62 × 62 × 86 Å. Ten independent docking runs were performed for each ligand using AutoDock Vina to ensure the reproducibility of binding conformations.^31^

The *in silico* assessment of absorption, distribution, metabolism, and elimination (ADME) parameters was performed using the SwissADME platform.^32^

Oleic acid was selected as the molecular marker for computational modeling due to its quantitative predominance in *andiroba* oil (∼52–56% of total fatty acids) and its well-documented anti-inflammatory activity, which is mediated through the inhibition of NF-kB and iNOS.^8,10,33,34^ Moreover, the availability of high-quality crystallographic data (CCDC No. 1226004) enabled robust docking and surface analyses, unlike limonoids, which are also present in *andiroba* but in minor fractions and lack complete structural data sets.

### 2.5 Nitric Oxide Release Assay

To evaluate anti-inflammatory activity, unloaded and *andiroba* oil-loaded sodium alginate sponges (10 mg) were dissolved in 1 mL of complete Dulbecco’s modified Eagle’s medium (DMEM) (Gibco, Grand Island, NY, USA), vortexed until solubilized, centrifuged at 1500 rpm for 10 min at 4 °C (BR4i Jouan, Saint-Herblain, France), and filtered through 22 μm sterile membrane filters (Kasvi, Pinhais, PR, Brazil).

Anti-inflammatory activity was assessed by measuring the ability of the sponges to modulate macrophage activation through nitric oxide production. Murine RAW 264.7 macrophages (1 × 10^5^ cells/well) were seeded in 96-well plates with DMEM supplemented with 10% fetal bovine serum (FBS) (Gibco, Paisley, U.K.), 1 mM pyruvic acid (Gibco, Grand Island, NY, USA), 0.25 μg/mL amphotericin B (Gibco, Grand Island, NY, USA), 10 U/mL penicillin (Gibco, Grand Island, NY, USA), and 10 μg/mL streptomycin (Gibco, Grand Island, NY, USA). After overnight incubation (37 °C, 95% humidity, 5% CO_2_), nonadherent cells were removed, and adherent cells were treated with sponge supernatants at 500, 250, 100, and 50 μg/mL. Cells were then stimulated with lipopolysaccharide (LPS; 1 μg/mL) for 24 h. Untreated LPS-stimulated cells were used as the negative control for the nitric oxide assay, and LPS-stimulated cells treated with IC_50_ (1 μM) of dexamethasone were used as the positive control.

To evaluate nitric oxide release, culture supernatants were collected 24 h after LPS stimulation. Aliquots of 50 μL were mixed with 50 μL of Griess reagent and incubated for 10 min protected from light, and absorbance was measured at 550 nm using a BioTek ELx808 microplate reader (BioTek, Winooski, USA). The results were expressed as a percentage of nitrite production relative to the control.^35^

### 2.6 Cytotoxicity Assay

Cytotoxicity was evaluated using the MTT assay at the same concentrations used in the NO assay (500, 250, 100, and 50 μg/mL) in RAW 264.7 macrophages.^36,37^ Following nitric oxide quantification, 90 μL of fresh DMEM and 10 μL of an MTT solution (5 mg/mL) were added to each well. After 3 h of incubation at 37 °C, supernatants were discarded, and the resulting formazan crystals were dissolved in 100 μL of dimethyl sulfoxide (DMSO) (ISOFAR, Duque de Caxias, RJ, Brazil). Cells grown in the culture medium alone were used as the negative control. Absorbance was recorded at 550 nm, and cell viability was expressed as the percentage relative to that of the untreated control.

All biological experiments were performed in triplicate (*n* = 3), and results were expressed as the mean ± standard deviation. Statistical significance was evaluated using one-way ANOVA followed by Tukey’s post hoc test, adopting a significance level of *p* < 0.05 for all comparisons.

## 3 Results and Discussion

### 3.1 Structural Analysis and Surface Morphology

Figure 1 presents the XRD patterns of the unloaded and *andiroba-*loaded sponges. The diffractograms were used to assess the structural organization of the materials. The unloaded alginate sponge exhibits a characteristic amorphous pattern, emphasized by a broad diffuse halo between 2θ = 13 and 36°, a region now highlighted in the figure for clarity. This feature confirms the amorphous nature of the alginate-based matrices, which contributes to their flexibility and ability to accommodate oil molecules. Such behavior is well documented for natural polysaccharides, including sodium alginate, and reflects the predominance of disordered regions within the polymeric network.^13^ The absence of distinct crystalline peaks indicates that no ordered phases were formed, a desirable property for systems designed for controlled release of bioactive compounds, as it enhances structural flexibility and hydration capacity, favoring the diffusion of active molecules through the matrix. After incorporation of *andiroba* oil, the same amorphous halo remained without generating new crystalline reflections, suggesting that the oil does not induce ordered rearrangement or crystallization of the matrix. The slight reduction in halo intensity, visible in the figure, suggests partial compaction and localized reorganization of the polymer chains due to hydrophobic interactions between alginate and the lipophilic components of the oil.^38,39^

**Figure 1:**
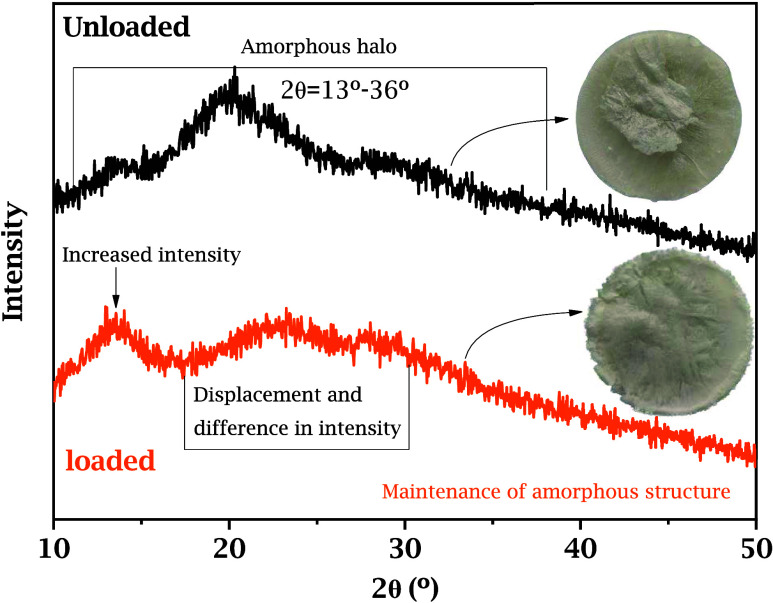
XRD diffractograms of the unloaded sodium alginate sponges (black) in the angular region of 2θ = 13–36° and *andiroba* oil-loaded sodium alginate sponges (orange) in the angular region of 2θ = 10–50°. The broad halo indicates that the amorphous structure is preserved after oil incorporation. Insets show representative images of the sponge morphology.

This is consistent with previous studies demonstrating that systems containing long-chain fatty acids, such as those present in *andiroba* oil (*e*.*g*., oleic acid), when dispersed in polymeric matrices, tend to remain disordered without promoting crystalline organization, as measured by XRD.^13,14,23^ This amorphous profile, even after loading of the polysaccharidic matrix with the oil, is highly desirable in pharmaceutical formulations intended for topical application. Amorphous structures provide greater matrix flexibility, enhance bioadhesion to the skin, and facilitate the diffusion of loaded bioactive compounds; however, their lower thermodynamic stability compared to crystalline forms can increase reactivity, potentially affecting the retention and stability of the incorporated bioactives.^14^

From a functional perspective, the maintenance of the amorphous structure, even after the addition of *andiroba* oil, reinforces the suitability of the alginate matrix as a topical delivery system. The lack of crystallinity favors the localized retention of *andiroba*’s bioactive limonoids (such as gedunin and andirobin) and fatty acids (such as oleic acid), which act as anti-inflammatory agents by inhibiting cytokines such as TNF-α and IL-1β.^10,33^ Therefore, the XRD analysis confirms that the sodium alginate matrix (alone and enriched with *andiroba* oil) has an amorphous nature, which is an expected and beneficial result for its use as a dressing for topical anti-inflammatory action.

SEM micrographs provide crucial structural information for understanding the functional behavior of sponges as bioactive dressings. Figure 2(a) shows the unloaded sodium alginate sponge displaying a highly porous, 3D morphology with relatively well-defined pores and thin, continuous septal walls. This architecture, interconnected pores and thin septa, is typical of freeze-dried hydrophilic polymeric matrices and is desirable for dressings because it favors the high absorption capacity of exudates, facilitates nutrient and gas diffusion, and allows cellular infiltration when applicable.^40,41^ The relative regularity of the pores also suggests that the emulsification of the oil within the aqueous solution of sodium alginate, followed by freeze-drying, was effective in maintaining a stable pore arrangement, which is associated with good moisture retention capacity and mechanical properties typical of alginate sponges used in dressings.^20,42^

**Figure 2:**
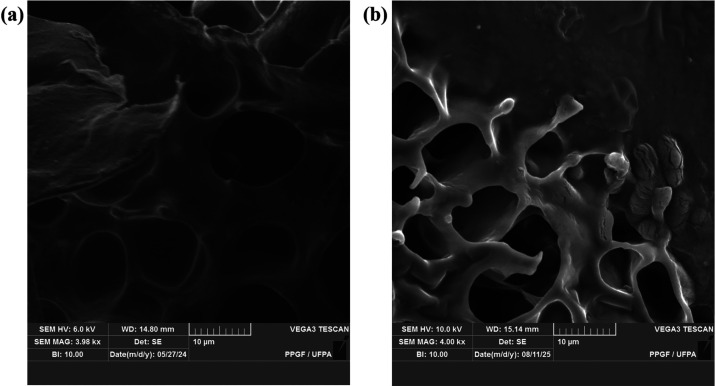
Morphology of unloaded sodium alginate sponge (a) and of *andiroba* oil-loaded sodium alginate sponge (b), taken by scanning electron microscopy with a scale bar of 10 μm.

In Figure 2(b), a clear microstructural change is observed when loading the alginate sponges with the oil: the pores appear more irregular, with membranous bridges and thicker walls, and areas where the pore edges appear as “harnesses” or recesses, when compared to the unloaded counterpart (Figure 2(a)). This denser appearance, with less-defined walls, is consistent with the incorporation of oils and lipophilic components into the polymer’s aqueous phase used in the production of the sponges. During emulsification of *andiroba* oil in the alginate aqueous solution and subsequent freezing/lyophilization, oil droplets or lipid films tend to form at the interfaces and can plasticize or refract the polymer chains, resulting in more flexible septa and less crystalline walls. This effect has already been described when hydrophobic substances are incorporated into hydrophilic matrices. There is a reduction in local apparent porosity, an increase in wall density, and the formation of lipid-rich microdomains.^13,21^ The presence of lamellar structures and small clusters in the right region of the micrograph in Figure 2(b) may reflect microclusters of oil or even residues from the drying process, a common event in lyophilized oil-in-water emulsion systems.^23^

From a functional perspective, these changes have significant consequences. Thicker walls and less-defined pores tend to reduce the initial rate of fluid absorption and slow down the diffusion of soluble compounds, favoring an even slower release of the lipophilic ones.^42^ This feature is particularly promising for anti-inflammatory dressings: the oil retained within the septum acts as a lipid reservoir, enabling a controlled and gradual release of *andiroba* fatty acids and limonoids. This mechanism helps maintain a localized therapeutic effect while minimizing the risk of systemic absorption.^13,14^ On the other hand, the reduction in poral opening can slightly reduce the maximum exudate absorption capacity; therefore, the balance between porosity and lipid load should be optimized for the intended clinical application of the dressing (low-exudative vs moderate-exudative wounds).

In terms of biocompatibility and tissue-cell interaction, relatively smooth and continuous surfaces (as observed in Figure 2(b)) may decrease the initial physical adhesion of cells that prefer rough topographies. Still, *andiroba*’s bioactive compounds (fatty acids, limonoids) may modulate the local inflammatory response and promote repair processes, offsetting any adverse mechanical effects. Furthermore, alginate itself is known for its biocompatibility and ability to maintain cell viability.^40^ At the same time, the incorporation of the oil, as long as it does not pose any toxicological risk, adds pharmacological functionality to the biomaterial.

The micrographs also suggest that control of the emulsification process (droplet size), freezing conditions, and lyophilization parameters is crucial for the final microstructure. Adjustments of steps allow modulating porosity, septa thickness, and oil distribution, which in turn regulate mechanical properties, exudate retention capacity, and bioactive release kinetics.^20,43^ In summary, the comparison between both panels in Figure 2(a,b) indicates that the incorporation of *andiroba* oil predictably alters the sponge’s microarchitecture, giving it a denser morphology that is potentially favorable for the modified release of anti-inflammatory compounds.

Quantitative analysis of the SEM images using ImageJ software revealed average pore diameters of approximately 5.2 μm for the unloaded sponges and 4.6 μm for the *andiroba-*loaded sponges, together with total porosities of 67.2% and 77.8%, respectively. The increase in porosity upon oil incorporation can be attributed to the presence of dispersed lipid domains acting as temporary templates during freezing, which promote the formation of additional voids after lyophilization. This more open and interconnected architecture is advantageous for fluid absorption and may facilitate cell penetration and nutrient exchange, enhancing the potential of the sponges for wound-healing applications.

The overall morphology of the sponges, characterized by high porosity, uniform pore distribution, and structural integrity, suggests mechanical and swelling behaviors comparable to those reported for similar sodium alginate dressings. Literature data indicate elastic moduli between 20 and 50 kPa and swelling ratios of 700–900%, values that ensure adequate flexibility, resilience, and fluid absorption for topical applications.^40,44,45^ These reported parameters are consistent with the observed microstructure and visual stability of the present formulations, supporting their functional suitability as wound-dressing biomaterials.

Figure S1 (Supporting Information) presents the EDS analysis performed to complement the morphological data obtained by SEM and to confirm the elemental composition of the sponges before and after the incorporation of *andiroba* oil. For the unloaded sponge, the spectrum showed a predominance of oxygen (48.8%) and carbon (39.8%), accompanied by a characteristic signal of sodium (10.8%), an element inherent to the structure of sodium alginate. After the incorporation of the oil, a significant increase in the relative content of carbon (51.9%) and nitrogen (from 0.6% to 5.6%) was observed, followed by a reduction in the oxygen content (31%). This increase in the carbon signal is directly associated with the presence of triglycerides and fatty acids present in *andiroba* oil, while the presence of nitrogen may be related to small fractions of amides and natural alkaloids present in the lipophilic extract, as previously described in the literature for *C. guianensis*.^7,14,46–48^ These results consistently confirm that the oil was effectively incorporated and distributed within the polymer matrix. Although XPS can provide additional information on surface chemical states, EDS was sufficient to demonstrate the presence and retention of oil in the sponge structure, therefore confirming our research hypothesis.

The amorphous configuration of alginate chains provides greater free volume and segmental mobility, which enhances the diffusion of small lipophilic molecules trapped within the matrix. This disordered arrangement enables the formation of transient microchannels during hydration, allowing for controlled release governed by polymer relaxation and water uptake kinetics.^40,42^ Such structural flexibility is critical for achieving sustained topical delivery without burst effects.

The structural and morphological characteristics of the alginate sponges, particularly their amorphous nature and interconnected porous network, are consistent with a diffusion-controlled release mechanism for the incorporated *andiroba* oil. Similar alginate matrices have been reported to exhibit gradual release of lipophilic molecules governed by polymer relaxation and water uptake dynamics.^40,49^ These features suggest the potential of our *andiroba* oil-loaded sponges to serve as a modified-release topical dressing, providing sustained local effects while minimizing systemic exposure.

### 3.2 Vibrational Analysis by Fourier Transform Infrared (FT-IR) Spectroscopy

FT-IR spectroscopic analysis was performed to characterize the *andiroba* oil functionally and to identify its main chemical groups responsible for the biological activity as well as interaction with the sodium alginate polymeric matrix. The FT-IR absorption spectrum obtained herein reveals the vibrational bands characteristic of lipid compounds, consistent with its fatty-acid-rich composition, as shown in Figure 3.

**Figure 3:**
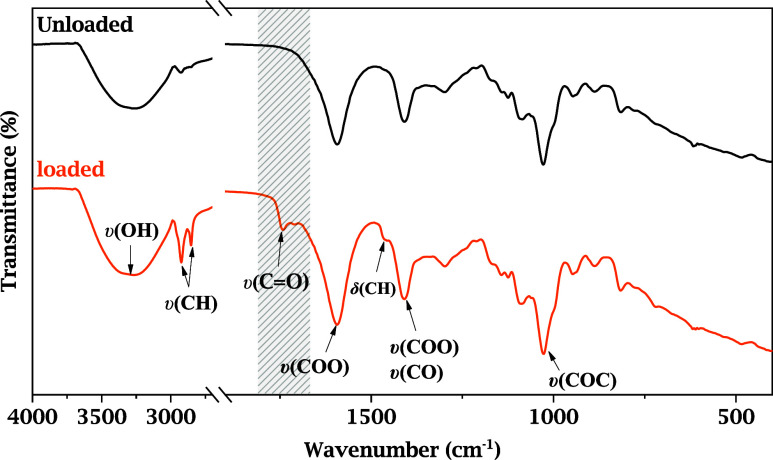
Overlay of FT-IR spectra, obtained in transmittance mode, of unloaded sodium alginate sponges (black) and *andiroba* oil-loaded sodium alginate sponges (orange).

The presence of a broad band in the spectral region of ∼3300 cm^–1^ is attributed to the stretching vibration due to hydroxyl units (OH), strongly associated with the presence of physically absorbed water in the sample. This band is also typical of *in nature* oils (as happens with *andiroba* used in this work). It may be related to hydrogen-bonding interactions, often recorded in minimally processed oils or oils stored under ambient humidity conditions.^9,14^

Among the main signals recorded in the FT-IR absorption spectrum of loaded sponges, the band at about 1743 cm^–1^ stands out, attributed to the stretching vibration of carbonyl units (C=O) from esters, typical of triglycerides present in vegetable oils. These esters result from fatty acids, such as oleic, linoleic, and palmitic, bound to glycerol molecules, composing the majority of the lipid fraction of *andiroba* oil.^9,13,40^

The interpretation of the FT-IR spectra is supported by reference data available for the *andiroba* oil. Silva et al.^47^ reported absorption bands at approximately 1714 cm^–1^ and 1812 cm^–1^ for the oil of *C. guianensis*. Silva et al.^48^ observed bands at 1746 cm^–1^ and 1464 cm^–1^, related to aliphatic ester and C–H bending vibrations, respectively. In our sponge formulations, minor shifts toward lower wavenumbers were observed for these characteristic bands, which may be attributed to hydrogen bonding and electrostatic interactions between the alginate matrix and triglyceride composing the oil.

In the spectral range from 2850 to 2924 cm^–1^, absorption bands attributed to symmetric and antisymmetric stretching vibrations of CH_2_ groups, widely present in the long hydrocarbon chains of saturated and unsaturated fatty acids, are noticed. These signals reinforce the presence of extensive and disorderly aliphatic chains, typical of amorphous systems with high molecular flexibility.^41^

Additionally, an IR absorption band around 1593 cm^–1^ is observed, which is attributed to the antisymmetric stretching vibration of the carboxylate group (COO) or the presence of conjugated C=C–H double bonds in unsaturated fatty acids, such as oleic acid.^50^ The presence of this band also suggests indirect contribution of intermolecular interactions involving polar functional groups and, possibly, traces of mild oxidation or hydration of the vegetable oil.^9,37^

The absorption band near 1408 cm^–1^ can be attributed to a combination of symmetric stretching of the carboxyl group (COO),^37^ suggesting the presence of partially ionized or free fatty acids, and stretching of the C–O bond in esters, characteristic of triglycerides.^51^ This overlap of vibrational motions is common in complex vegetable oils and reinforces the presence of a natural mixture of free and esterified lipids in *andiroba* oil.^9,52^

The absorption band observed at about 1026 cm^–1^ corresponds to the stretching vibration from the C–O–C bond,^41^ especially associated with ester groups formed by the esterification of fatty acids with glycerol. This signal reinforces the predominance of triglycerides as the main components of the *andiroba* oil. These findings are in agreement with the literature, which describes *andiroba* oil as a complex plant extract containing mainly esterified fatty acids, in addition to secondary compounds due to the presence of limonoids (gedunin, andirobin), with recognized anti-inflammatory and healing actions.^10,23^

Table 1 summarizes the main IR absorption bands of unloaded and *andiroba* oil-loaded alginate sponges, highlighting characteristic alginate vibrations and the additional lipid-related bands associated with oil incorporation.

**Table 1:** Main IR Absorption Bands of Unloaded and *Andiroba* Oil-Loaded Alginate Sponges and Their Vibrational Assignments

wavenumber (cm^–1^)	assignments	unloaded	loaded
∼3300	ν(O–H) stretching (hydroxyl groups of alginate/polysaccharides)	strong, broad	strong, slightly intensified
2920–2850	ν(C–H) stretching (aliphatic chains of lipids)	very weak	clearly present/intensified
∼1740	ν(C=O) stretching of ester groups (triglycerides from *andiroba* oil)	absent	present (new band)
∼1620–1600	ν(COO^–^) asymmetric stretching (alginate carboxylates)	strong	strong, slight shift
∼1420	ν(COO^–^) symmetric stretching	present	present
1360–1370	δ(CH) bending	weak	more evident
1240–1260	ν(C–O)/ν(COO) (oil + alginate contributions)	present	intensified
1080–1030	ν(C–O–C) glycosidic and ring vibrations	strong	strong
∼990–1000	ν(C–O–C) polysaccharide skeletal vibration	present	present
820–900	M/G block vibrations (mannuronic/guluronic units)	present	present

### 3.3 Hirshfeld Surface Analysis

Hirshfeld surface analysis was performed to understand, at the molecular level, the types and intensities of intermolecular interactions present in oleic acid, *i*.*e*., one of the main bioactive compounds of *andiroba* oil. This theoretical approach allows a detailed evaluation of interatomic contacts, revealing regions of potential interaction through van der Waals forces, hydrogen bonds, and electrostatic contacts that are directly related to the biological activities of oleic acid, especially its use as an anti-inflammatory bioactive ingredient.

In Figure S2­(a) (Supporting Information), the Hirshfeld surface mapped by *d*_norm_ is observed, which highlights regions of intense interaction through the red spots located at the carboxylic ends of the molecule. These areas indicate strong O···H contacts, characteristic of hydrogen bonds, which are essential for the molecule’s interaction with biological targets, such as enzymes involved in classical inflammation pathways. The presence of these interaction zones favors the acid oleic affinity for protein catalytic sites, aiding in the selective inhibition of the inflammatory response.^53^

In Figure S2­(b), the shape index reveals alternating shades of blue and red in wavy regions throughout the molecule. These patterns indicate zones of steric complementarity, which are essential for molecular recognition in protein active sites. Although the oleic acid is predominantly aliphatic, this topological distribution may favor specific interactions based on 3D “fitting,” contributing to selective affinity with inflammatory enzymes. The presence of these structural domains reinforces the hypothesis that the anti-inflammatory activity of oleic acid may be related to steric and conformational blocking mechanisms in the catalytic sites of inflammatory proteins.^54^

In Figure S2­(c), the surface reveals regions of low (flat areas in light blue) and high (wavy areas in green and yellow) curvatures. The flat areas are characteristic of efficient van der Waals contacts, especially along the hydrocarbon chain, and indicate potential lipophilic interaction with cell membranes, which may facilitate the penetration of the oleic acid and its action at inflammatory sites, as described by Fonseca et al.^23^ for topical formulations containing *andiroba* oil.

Figure S2­(d) represents the voids generated in the crystal packing of oleic acid. Analysis reveals that approximately 70% of the structure’s volume is occupied by molecular voids, indicating a low-density and highly porous packing. This characteristic is advantageous for modified-release systems, as it facilitates the diffusion of small molecules, such as water and ions, and allows for a slower release of the active ingredient from alkyl alginate sponges. This structure may also favor interaction with biological fluids and tissue penetration, optimizing topical anti-inflammatory efficacy.^14^ Geometric parameters calculated from the surface indicate a surface area of 731.72 Å^2^ and a molecular volume of 198.61 Å^3^, with a globularity of 0.225, translating to an elongated, nonspherical structure. The asymmetry (asphericity) of 0.759 confirms this irregular morphology, which contributes to a high intermolecular contact area and versatility in interactions with different targets. These structural properties are consistent with bioactive compounds known to modulate inflammatory responses by targeting signaling pathways involved in inflammation.^8^

The two-dimensional (2D) fingerprint plots derived from Hirshfeld surface analysis provide a quantitative representation of the intermolecular contact distribution for the analyzed compound. Figure S3­(a) (Supporting Information) shows the complete interaction map, where all contact types collectively account for 100% of the surface contributions. The dominant interactions are hydrophobic H···H (86.5%), as shown in Figure S3­(b), which is characteristic of molecules enriched in long alkyl chains, such as fatty acids. Figure S3­(c) highlights O···H/H···O hydrogen-bonding interactions (10.7%), indicating the presence of polar regions capable of forming directional contacts relevant for molecular recognition. The C···H/H···C contributions, presented in Figure S3­(d), represent only 1.8% of the total interactions, reflecting their limited steric and energetic significance. Overall, the fingerprint profile confirms that although oleic acid is predominantly hydrophobic, it still contains functional regions capable of engaging in polar interactions, which may support its affinity for biological targets associated with inflammatory pathways.

Hirshfeld surface analysis, therefore, reveals that the oleic acid presents a combination of reactive polar zones and nonpolar contact regions, which favors molecular interactions with different biological targets. This structural duality may explain the anti-inflammatory efficacy of *andiroba* oil, already demonstrated in *in vitro* and *in vivo* assays, which revealed inhibition of nitric oxide production by activated macrophages and a reduction in the expression of inflammatory mediators.^8^ These findings contribute to the rational use of *andiroba* oil in topical pharmaceutical dressings due to its anti-inflammatory action.

### 3.4 Molecular Docking and Pharmacokinetic Properties

The evaluation of the molecular interaction of oleic acid, the main fatty acid in the *andiroba* oil^46^ with relevant inflammatory targets, was conducted through molecular docking studies, aiming to understand its possible anti-inflammatory modulation mechanisms. Three key proteins involved in the inflammatory process were selected: IkBα/NF-kB inhibitory complex (PDB: 1IKN), and iNOS (PDB: 6DN6),^55^ of recognized importance in the mediation and maintenance of the inflammatory response.

NF-kB acts as a central transcription factor in the expression of pro-inflammatory genes, being kept inactive in the cytoplasm by binding to IkBα. Figure S4­(a) (Supporting Information) shows the molecular coupling with the macromolecule and reveals that oleic acid presented the best binding free energy with the IkBα/NF-kB complex (−5.75 kcal/mol) and an inhibition constant (*K*_i_) of 61.34 μM. The fatty acid establishes hydrophobic interactions (alkyl and π-alkyl) with residues, such as LYS249, VAL251, and HIS245, and a hydrogen bond with GLU292, as shown in the 2D interaction map in Figure S4­(b). This affinity suggests that oleic acid may stabilize the inhibitory interaction between IkBα and NF-kB.^33,56^

NF-kB activation occurs through various factors, including the recognition of pathogen-associated molecular patterns, such as LPS, or the activation by cytokines like IL-1β and TNF, which triggers a phosphorylation cascade of intracellular proteins, including IkBα, responsible for inactivating NF-kB. Once translocated to the nucleus, NF-kB promotes the expression of proteins involved in the inflammatory process, such as iNOS.^55^ Stabilizing the interaction between IkBα and NF-kB may thus reduce the level of gene activation and prevent the exacerbated production of inflammatory mediators.

Figure S5­(a) (Supporting Information) shows the molecular docking of oleic acid with iNOS, where the binding free energy was −5.15 kcal/mol and *K*_i_ 167.41 μM. Figure S5­(b) shows that the interactions involved a combination of hydrogen bonding, hydrophobic contacts, and van der Waals forces. The carboxyl group of oleic acid formed hydrogen bonds with GLN143, GLY144, and LYS145, anchoring the ligand in the catalytic pocket. In addition, the aliphatic chain was stabilized by hydrophobic interactions (alkyl and π-alkyl) with residues such as LEU140, PRO150, PHE149, TYR141, TRP131, HIS142, LEU194, ALA190, and ARG192, consistent with the long nonpolar tail of the fatty acid. van der Waals contacts with ALA149, SER144, and GLY148 further reinforced the accommodation of the ligand. A minor unfavorable interaction with PRO147 was also observed, but did not compromise the overall stability of the complex.

Although nitric oxide is essential for the progression and resolution of inflammation at reasonable levels, overexpression of iNOS and increased production of this radical can assume a pro-inflammatory character.^57^ Regulation of iNOS is most effective at the expression level;^58^ thus, our data indicate that the compound acts on both NF-kB, one of the main transcription factors for the enzyme’s expression, and on iNOS itself, highlighting the sponge’s anti-inflammatory potential.

The *in silico* pharmacokinetic analysis of oleic acid demonstrates a suitable profile for topical applications, particularly in formulations designed to treat skin inflammation, as shown in Table 2. One of the most notable observations is its low transdermal permeation, as shown by a Log *K*_*p*_ of −2.6 cm/s. This parameter indicates that oleic acid tends to remain in the most superficial layers of the skin, favoring a more local action without the risk of systemic absorption.^42^ This characteristic is desirable for the development of anti-inflammatory dressings, as it reduces potential side effects and increases the safety for topical use.

**Table 2:** *In Silico* ADME Results for the Oleic Acid, the Main Biomarker of *Andiroba Oil*

physicochemical properties	
molecular weight (g/mol)	282.46
TPSA (Å^2^)	37.3
Lipophilicity	
Log *P*_o/w_ (SILICOS-IT)	5.95
Water solubility	
Log *S* (SILICOS-IT)	–5.39
solubility (mg/mL)	1.14 × 10^–3^
class	moderately soluble
Pharmacokinetics	
GI absorption	high
BBB permeant	no
P-gp substrate	no
CYP1A2 inhibitor	yes
CYP2C19 inhibitor	no
CYP2C9 inhibitor	yes
CYP2D6 inhibitor	no
CYP3A4inhibitor	no
Log *K*_*p*_ (skin permeation) (cm/s)	–2.6
Druglikeness	
Lipinski	yes, 1 violation: M LOG* P* > 4.15
Ghose	no, 1 violation: M LOG *P* > 5.6
Veber	no, 1 violation: Rotors >10
Egan	no, 1 violation: M LOG *P* > 5.88
Muegge	no, 1 violation: X LOG* P*3 > 5
Bioavailability score	0.85
Medicinal chemistry	
PAINS	0
brenk	1 alert
leadlikeness	no, 2 violations: Rotors >7, X LOG *P*3 > 3.5
synthetic accessibility	3.07

Another important finding is that the oleic acid exhibits high gastrointestinal absorption, yet it is unable to cross the blood–brain barrier (BBB).^32^ Although oral absorption is not the primary focus of the study, this characteristic contributes to the understanding of the general pharmacokinetic behavior of the fatty acid. It reinforces the idea that topical use would not result in undesirable central effects. Furthermore, the compound is not a substrate for P-glycoprotein (P-gp), an efflux protein that typically limits the retention of therapeutic agents in cells. This means that when applied topically, the oleic acid tends to remain at the application site longer, enhancing its anti-inflammatory action.

From a metabolic perspective, oleic acid inhibits enzymes CYP1A2 and CYP2C9, indicating relevant biochemical activity, although it does not act on major hepatic metabolic pathways, such as CYP3A4. Because the intended application is onto the skin, enzyme inhibition has little systemic impact; however, it suggests a potential local, biologically active effect.

Although the oleic acid presents violations in some Druglikeness filters (such as Lipinski, Ghose, Veber, and Egan), these are related to its high lipophilicity (ranging from 4.2 to 7.6), which is expected for bioactives derived from vegetable oils. These characteristics favor penetration into superficial tissues and the formation of protective films, which are very useful in regenerative dressings.^42^

The oleic acid has no toxicity warnings, and the synthetic accessibility score indicates that it can be obtained and modified with relative ease. *In vitro* inflammatory-modulating activity combined with the low observed cytotoxicity reinforces its safety for topical formulations.

Despite its high lipophilicity (Log *P*_o/w_ = 5.95), oleic acid remains a suitable bioactive of *andiroba* oil to be formulated in sodium alginate sponges, which offer a modified release. The low water solubility (Log *S* = −5.39) reinforces this need but does not limit its use. Therefore, the pharmacokinetic profile supports the topical use of *andiroba* oil, highlighting its interesting properties as a promising candidate for dressings with localized anti-inflammatory action.

However, the ADME profile provides valuable information about the systemic behavior of bioactive molecules; its interpretation in this study must be framed within the purpose of a formulation developed for topical use. In topical applications, systemic absorption is generally limited by the epidermal barrier, which avoids the compound’s exposure to hepatic metabolism and, consequently, to interaction with cytochrome P450 (CYP) complex enzymes. Thus, although oleic acid, the main constituent of *andiroba* oil, shows potential to inhibit CYP isoforms *in vitro*, these effects are less relevant in cutaneous formulations, where the therapeutic action occurs predominantly locally, limiting any systemic pharmacokinetic effects. Previous studies have corroborated that systems based on fatty acids applied topically have low systemic bioavailability and minimal interference with hepatic drug metabolism.^42,59,60^ Therefore, in the context of this work, the ADME profile complements the understanding of the compound’s molecular interactions and overall safety. Still, it does not constitute a limiting factor for its application as a topical dressing.

### 3.5 Nitric Oxide Release Assay and Cytotoxicity

The inflammatory response and cytocompatibility of sodium alginate sponges were evaluated, respectively, by quantifying nitric oxide production in macrophages of the RAW 264.7 cell line stimulated with LPS, and by a cell viability assay using the MTT method.

The *andiroba* oil-loaded sponge exhibited a dose-dependent anti-inflammatory effect, evidenced by a significant reduction in nitric oxide production at concentrations of 250 (*p* = 0.0002) and 500 μg/mL (*p* < 0.0001). Furthermore, the reduction in NO at 500 μg/mL did not differ significantly from that of the positive dexamethasone control, as displayed in Figure 4. These findings align with previous studies by Inoue et al.^61^ and Higuchi et al.,^62^ who showed that *andiroba* oil effectively reduced nitric oxide levels in LPS-activated macrophages, confirming its anti-inflammatory and wound-healing properties. In addition, data from Monteiro et al.^14^ showed that an *andiroba* oil-based nanoemulsion exhibits wound-healing potential, with greater keratinocyte migration compared to pure oil in the cell-migration assay. Although *andiroba* oil has already been incorporated into various biomaterials, including emulgels,^23^ and wound-dressing films,^48^ studies evaluating its anti-inflammatory properties when embedded in these matrices remain limited. Oleic acid is known for its anti-inflammatory activity, including the downregulation of iNOS expression and nitric oxide synthesis in macrophages stimulated by LPS.^63^ It also inhibits the NF-kB signaling pathway by enhancing SIRT1 activity.^64^ Our molecular docking analysis revealed interactions between the compound and both the NF-kB pathway and the iNOS enzyme (key regulators of NO production), supporting the hypothesis that the *andiroba* oil in the sponge modulates these inflammatory pathways. This is consistent with the significant reduction in nitric oxide observed in our *in vitro* experiments.

**Figure 4:**
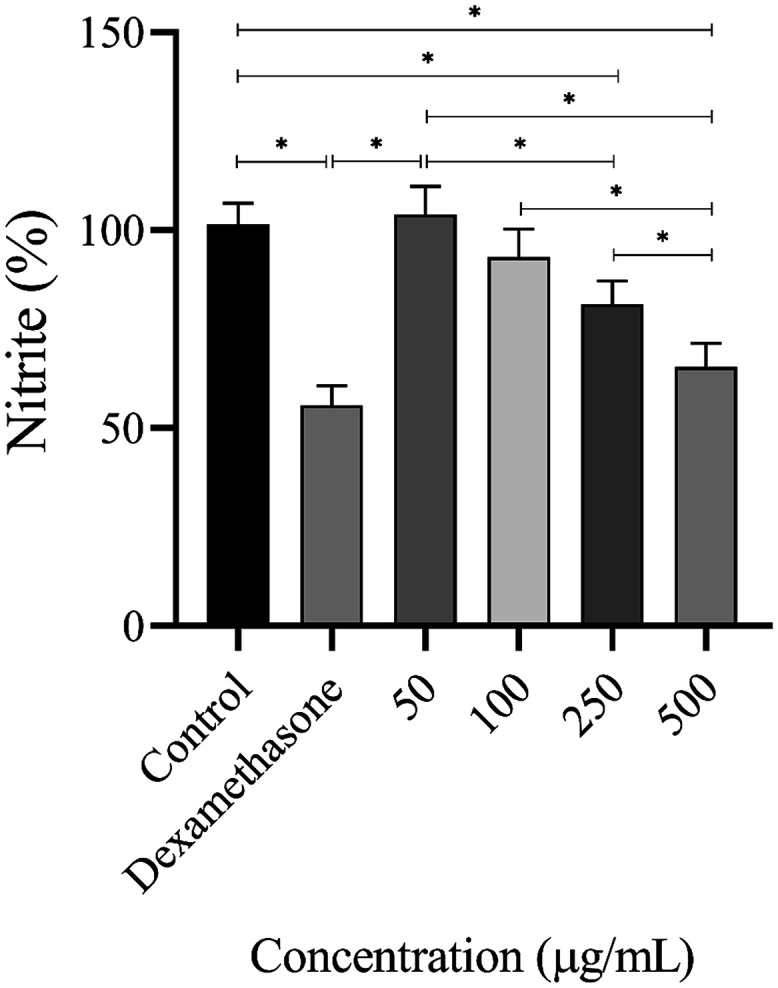
Percentage of nitrite measured after contact of RAW 264.7 cells with *andiroba* oil-loaded sodium alginate sponges, in comparison to the negative control (untreated LPS-stimulated cells) and positive control (1 μM of dexamethasone). Parametric ANOVA determined differences between each group. Data are presented as mean ± SEM of triplicate wells and * *p* ≤ 0.05 compared to the control group.

Taken together, the data indicate that incorporating *andiroba* oil into the sodium alginate matrix maintains the biomaterial safety profile and adds to its anti-inflammatory action *in vitro*. These features make the sponges a promising candidate for the development of dressings for inflammatory processes (*e*.*g*., wound healing and other chronic skin lesions).

Figure 5(a,b) shows that both the unloaded alginate sponges and the *andiroba* oil-loaded alginate sponges exhibited low cytotoxicity at concentrations of 50, 100, 250, and 500 μg/mL, respectively. Cell viability of unloaded sponge remained above 90% at all concentrations tested, which is in agreement with the recognized biocompatibility of sodium alginate, a natural polymer widely used in biomedical formulations due to its low toxicity, gentle gelation capacity, favorable interaction, and its ability to interact favorably with living tissues.^15,40,65^ Similarly, the loaded sponges led to a cell viability above 90% at all concentrations, indicating that the incorporation of the oil does not compromise cellular integrity. These data corroborate previous studies that demonstrated the low toxicity of the main components of *andiroba* oil (*i.e*., oleic and linoleic fatty acids), as well as secondary compounds (*i.e*., limonoids and triterpenes), which have pharmacological properties without inducing significant deleterious effects on normal cells.^14,23^

**Figure 5:**
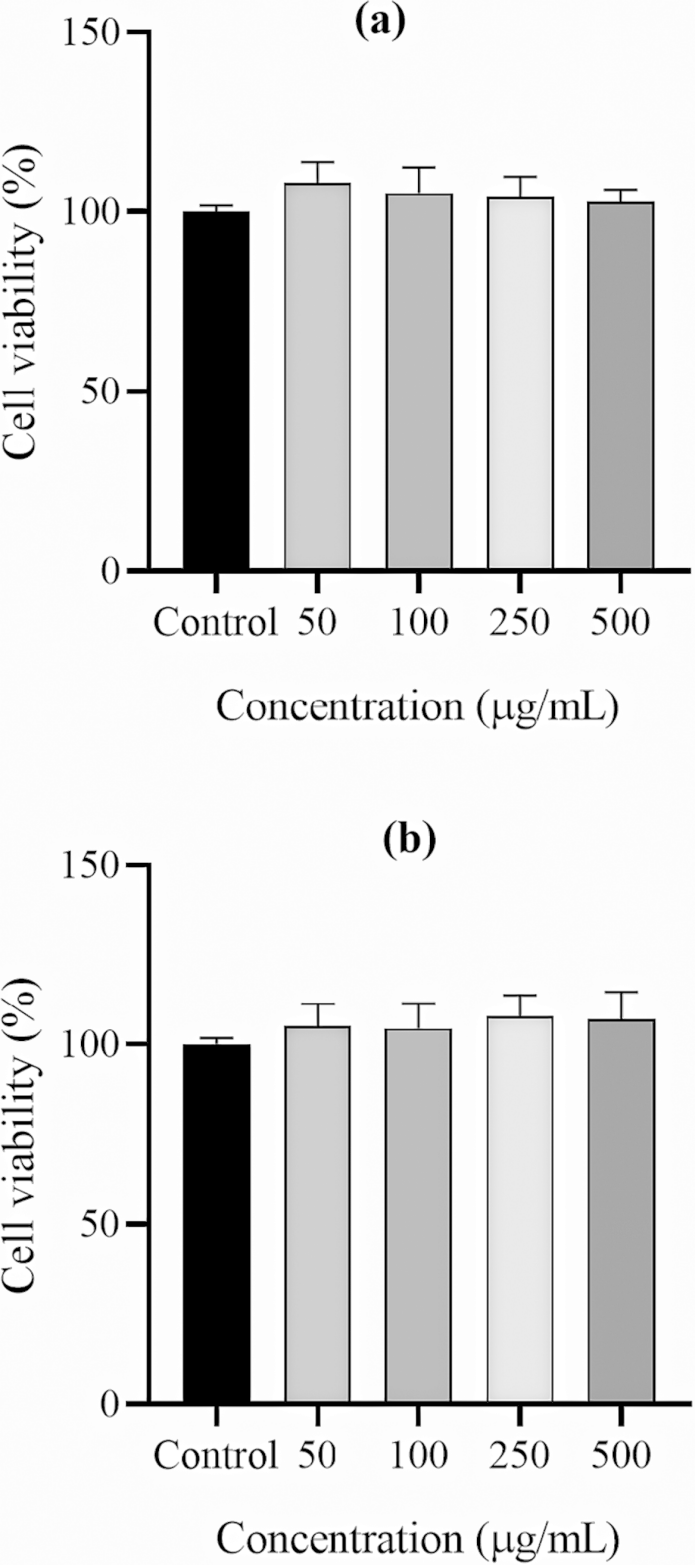
Cell viability of RAW 264.7 murine macrophages after 24 h incubation with unloaded (a) and andiroba oil-loaded (b) alginate sponges at concentrations of 50, 100, 250, and 500 μg/mL, assessed by the MTT assay. Cells cultured only in complete DMEM medium were used as the negative control. Data represent mean ± standard deviation (SD) of three independent experiments (*n* = 3). Statistical significance was determined using one-way ANOVA followed by Tukey’s post hoc test, with *p* < 0.05 considered significant.

The anti-inflammatory response observed for the *andiroba*-loaded sponge is consistent with the previously reported effects of *andiroba*-based formulations. Fonseca et al.^8^ reported a 45–60% reduction in nitric oxide production in LPS-stimulated macrophages treated with *andiroba* oil nanoemulsions at concentrations of 50–100 μg/mL. Similarly, Monteiro et al.^14^ demonstrated that emulgel formulations containing 2% *andiroba* oil decreased nitric oxide levels by approximately 55%, with no significant cytotoxicity. Seron da Fonseca et al.^23^ described comparable inhibition of nitric oxide release (≈ 50%) using polymeric nanocarriers loaded with the oil. In our study, the alginate sponge exhibited a comparable inhibitory response while maintaining a cell viability above 90%, confirming the biological compatibility and anti-inflammatory potential of the system. Furthermore, the solid, amorphous, and highly porous matrix of alginate is expected to sustain local retention and promote a modified release of the oil constituents, which may enhance and extend the anti-inflammatory action at the site of application. These results place the performance of the present sponge within the same efficacy range reported for other *andiroba*-based biomaterials and phytochemical dressings, reinforcing its functional suitability as a topical wound dressing.

## Conclusions

This study demonstrated that the incorporation of *andiroba* oil into polymeric alginate sponges yields a biomaterial with relevant anti-inflammatory activity suitable for topical application. XRD analyses confirmed the amorphous character of the matrix after the oil was loaded, a desirable characteristic for modified-release systems. SEM analysis revealed a highly porous and interconnected 3D architecture of the unloaded sodium alginate sponges. In contrast, the *andiroba* oil-loaded sponges exhibited morphological changes, including thicker and less-defined pore walls, irregular pore shapes, and localized denser regions, features attributed to the incorporation of the *andiroba* oil into the polymeric network. EDS analysis further confirmed the elemental signature of *andiroba* oil within the polymeric matrix, supporting the successful incorporation of the bioactive phase into the alginate sponge structure. FT-IR spectroscopy revealed the functional groups typical of *andiroba* fatty acids (*i*.*e*., oleic acid), indicating chemical interactions with the alginate matrix. Hirshfeld surface analysis and molecular docking with oleic acid indicated predominant hydrophobic interactions and hydrogen bonds with strategic inflammatory targets such as the IkBα/NF-kB complex and iNOS. These results suggest that the anti-inflammatory action occurs primarily through modulation of the NF-kB pathway and inhibition of the expression of enzymes involved in inflammatory processes. The pharmacokinetic profile obtained by *in silico* ADME tools showed low skin permeation (Log *K*_*p*_ = −2.6 cm/s), no interaction with P-glycoprotein, and no penetration into the BBB, reinforcing the safety profile for topical use and local retention of the oleic acid, a major component of the *andiroba* oil. *In vitro* biological assays demonstrated that the sponges containing *andiroba* oil significantly reduced nitric oxide production in RAW 264.7 macrophages stimulated with LPS, especially at concentrations of 250 and 500 μg/mL, without compromising cell viability, confirming that the addition of the oil enhanced anti-inflammatory activity. Thus, oil-loaded sponges represent a promising platform for the development of bioactive dressings for localized anti-inflammatory applications. In addition to leveraging an Amazonian herbal resource, the proposed biomaterial combines biocompatibility, structural stability, and pharmacological efficacy, representing an innovative and sustainable strategy for the treatment of inflamed wounds and chronic skin lesions.

## Supplementary Material



## Notes

Additional data related to the physicochemical and molecular characterizations of the developed sodium alginate sponges. It comprises elemental distribution analyses by EDS of loaded and unloaded sponges (Figure S1), Hirshfeld surface analyses, and 2D fingerprint plots of oleic acid (Figures S2 and S3), as well as molecular docking studies of oleic acid with the IkBα/NF-kB complex and iNOS. Additionally, three-dimensional binding conformations and two-dimensional interaction maps are provided in (Figures S4 and S5). This material supports and complements the results presented in the main text of the manuscript (PDF)
