# Supplementary material for: Physicochemical Characterization and In Vitro Anti-Inflammatory Assessment of Novel Sodium Alginate Sponges Loading Andiroba Oil (Carapa guianensis Aubl.) for Skin Dressings
Source: ACS Omega. 2026 Feb 4;11(6):9937–49. doi: 10.1021/acsomega.5c10665 (PMC12917710; doi:10.1021/acsomega.5c10665)
Supplement: Supplementary file 1 [file ao5c10665_si_001.pdf]

Supporting Information

Physicochemical characterization and *in vitro* anti-inflammatory assessment of novel sodium alginate sponges loading *andiroba* oil (*Carapa guianensis* Aubl.) for skin dressings

Marinaldo V. de Souza Junior[§,#], Jad Lorena F. Simplicio[§], Fernanda F. Costa[⊥], Aramys S. Reis[⊥], Eliana B. Souto[#*], Adenilson O. dos Santos[§], and Francisco F. de Sousa[§,†*]

[§]Center for Sciences of Imperatriz, Federal University of Maranhão – UFMA, 65900-410, Imperatriz, MA, Brazil.
[#]UCD School of Chemical and Bioprocess Engineering, University College Dublin, Belfield, Dublin 4, D04 V1W8, Ireland.
[⊥]Laboratory of Pathophysiology and Therapeutic Research, Center for Social Sciences, Health and Technology, Federal University of Maranhão, Imperatriz, MA, 65900-410, Brazil.
[†]Institute of Exact and Natural Sciences, Federal University of Pará – UFPA, 66075-110, Belém, PA, Brazil.

Corresponding authors.

[*]Eliana B. Souto, UCD School of Chemical and Bioprocess Engineering, University College Dublin, Belfield, Dublin 4, D04 V1W8, Ireland, eliana.souto@ucd.ie

[*]Francisco F. de Sousa, Institute of Exact and Natural Sciences, Federal University of Para – UFPA, 66075-110, Belém, PA, Brazil, ffs@ufpa.br

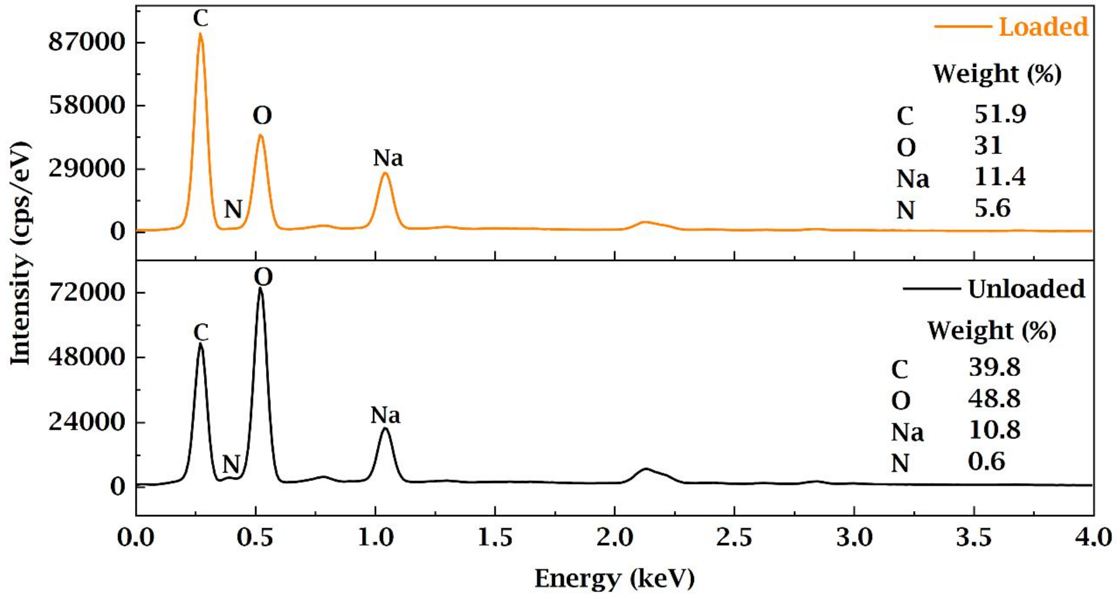

Fig. S1. Elemental distribution by EDS in sponges loaded with *andiroba* oil and unloaded sponges. For better interpretation, the orange lines represent loaded sponges with *andiroba* oil, and the black lines represent unloaded sponges.

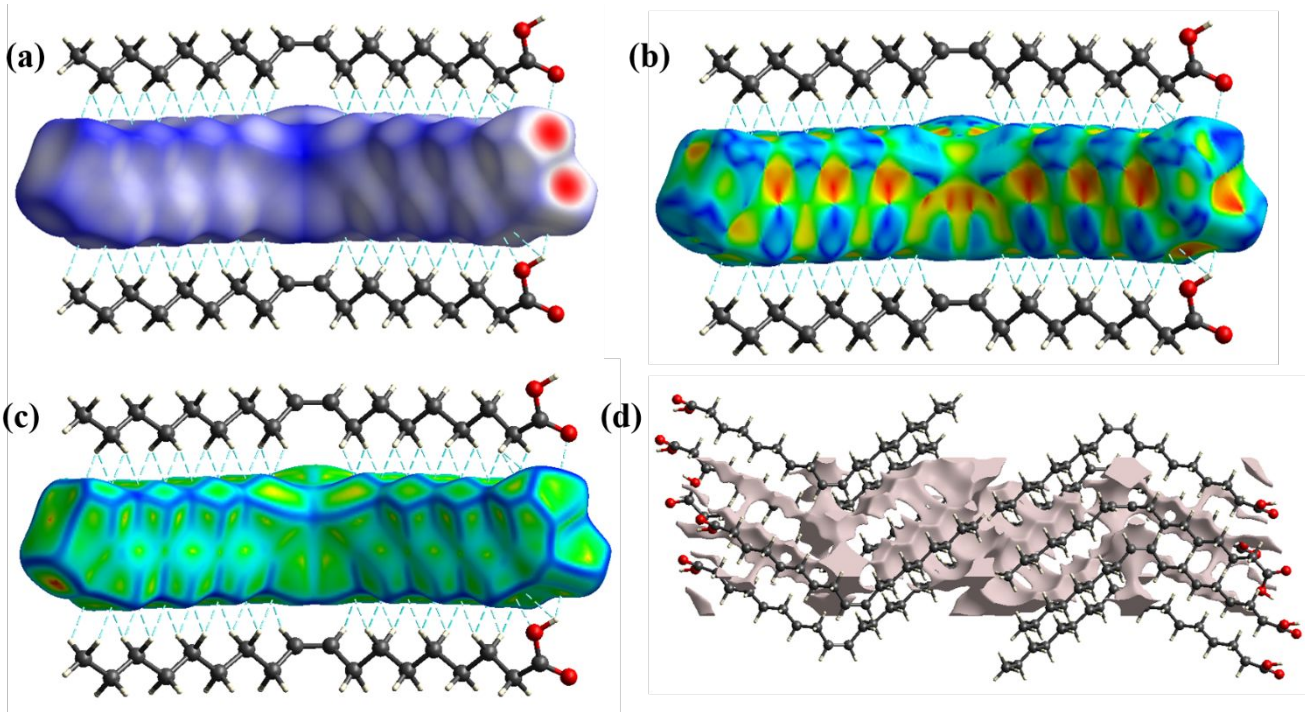

Fig. S2. Hirshfeld surfaces of oleic acid mapped according to (a) d_{norm}, (b) shape index, (c) curvature, and (d) voids.

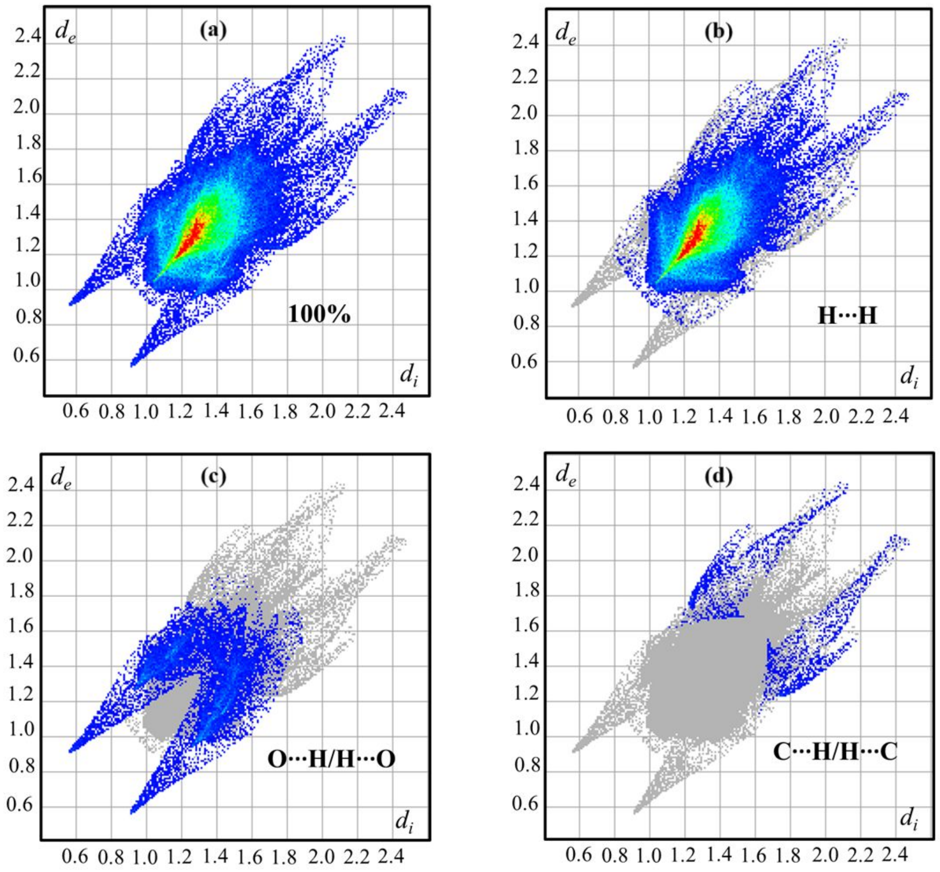

Fig. S3. (a) Full 2D fingerprint plot for the oleic acid molecule and specific fingerprint plots of interactions: (b) H···H = 86.5%, (c) O···H/H···O = 10.7%, and (d) C···H/H···C = 1.8%.

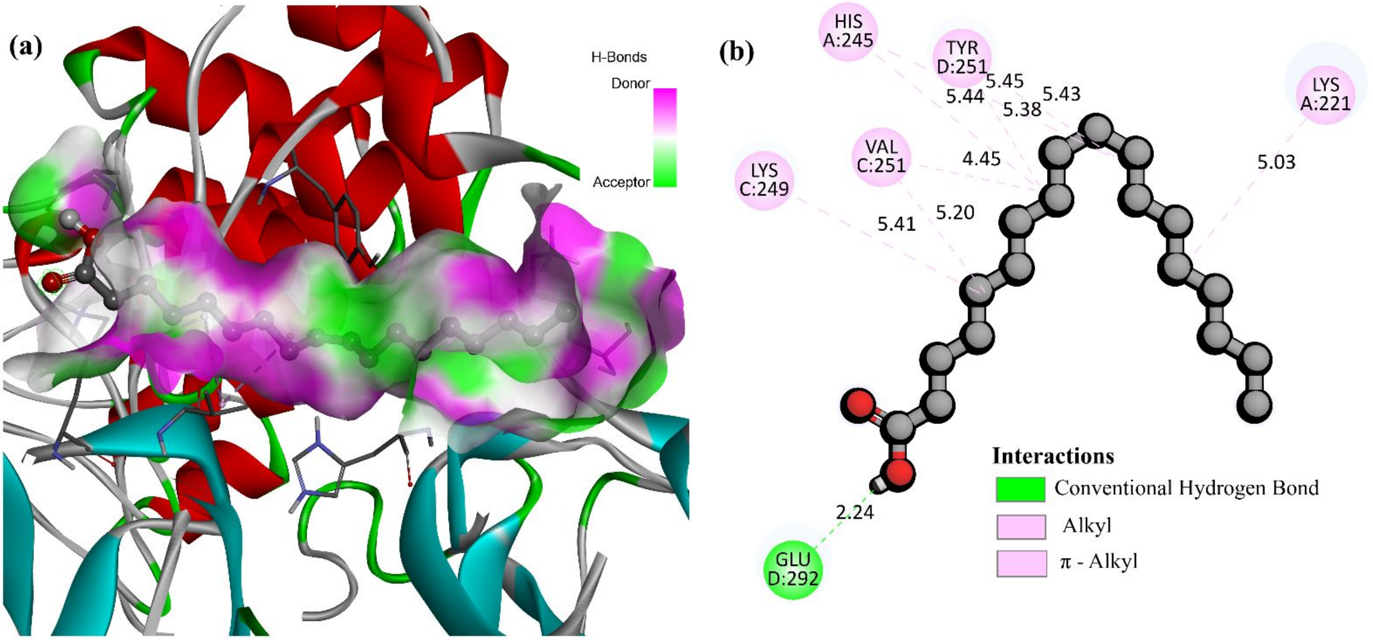

Fig. S4. (a) Molecular docking analysis of the IkBα/NF-kB complex with oleic acid, illustrating the predicted binding conformation. (b) Two-dimensional interaction map showing the specific amino acid residues involved in the binding of oleic acid to the IkBα/NF-kB interface.

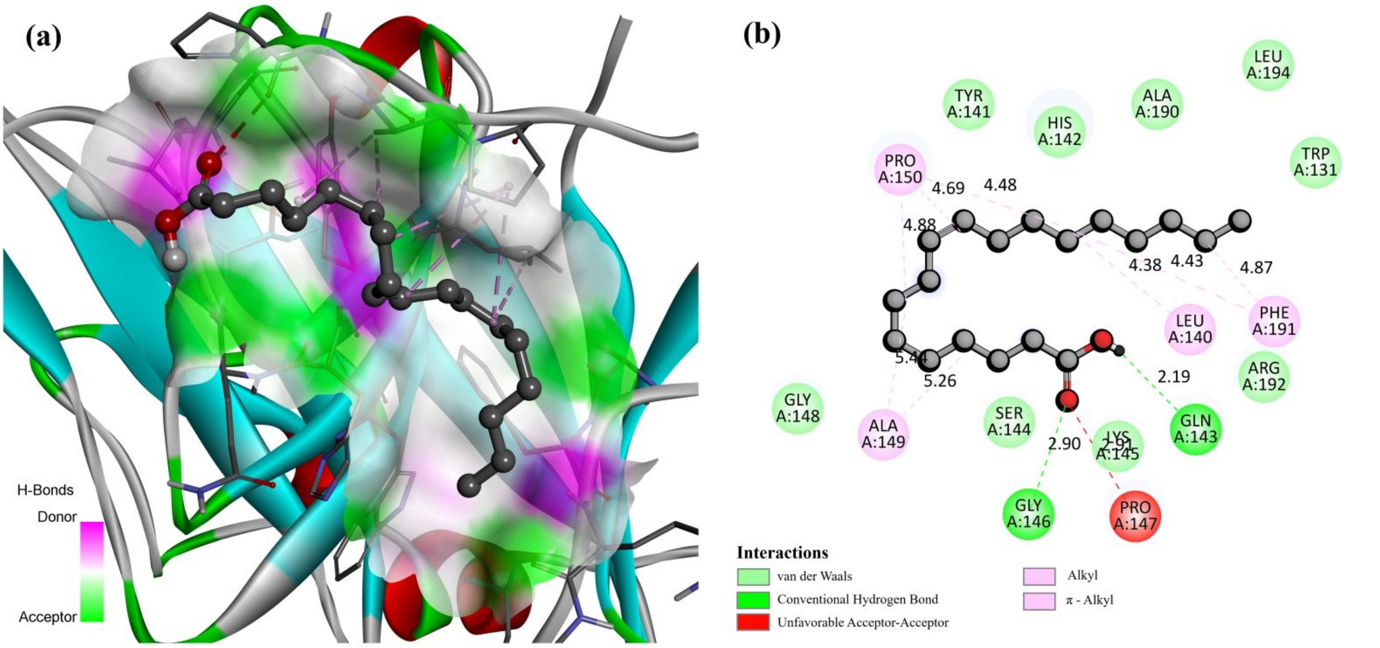

Fig. S5. (a) Molecular docking analysis of the iNOS with oleic acid, illustrating the predicted binding conformation. (b) Two-dimensional interaction map showing the specific amino acid residues involved in the binding of oleic acid to the iNOS interface.
